# Effect evaluation of a heated ambulance mattress-prototype on body temperatures and thermal comfort - an experimental study

**DOI:** 10.1186/s13049-014-0043-5

**Published:** 2014-08-08

**Authors:** Jonas Aléx, Stig Karlsson, Britt-Inger Saveman

**Affiliations:** 1 Department of Nursing and associated to Arctic Research Centre, Umeå University, Umea, SE-901 87, Sweden; 2 Department of Nursing, Umeå University, Umea, SE-901 87, Sweden; 3 Department of Nursing, Center for Disaster Medicine and associated to Arctic Research Centre, Umea University, Umea, SE-901 87, Sweden

**Keywords:** Thermal comfort, Cold discomfort, Cold exposure

## Abstract

**Background:**

Exposure to cold temperatures is, often, a neglected problem in prehospital care. One of the leading influences of the overall sensation of cold discomfort is the cooling of the back. The aim of this study was to evaluate the effect of a heated ambulance mattress-prototype on body temperatures and thermal comfort in an experimental study.

**Method:**

Data were collected during four days in November, 2011 inside and outside of a cold chamber. All participants (n = 23) participated in two trials each. In one trial, they were lying on a stretcher with a supplied heated mattress and in the other trial without a heated mattress. Outcomes were back temperature, finger temperature, core body temperature, Cold Discomfort Scale (CDS), four statements from the state-trait anxiety – inventory (STAI), and short notes of their experiences of the two mattresses. Data were analysed both quantitatively and qualitatively. A repeated measure design was used to evaluate the effect of the two mattresses.

**Results:**

A statistical difference between the regular mattress and the heated mattress was found in the back temperature. In the heated mattress trial, the statement “I am tense” was fewer whereas the statements “I feel comfortable”, “I am relaxed” and “I feel content” were higher in the heated mattress trial. The qualitative analyses of the short notes showed that the heated mattress, when compared to the unheated mattress, was experienced as warm, comfortable, providing security and was easier to relax on.

**Conclusions:**

Heat supply from underneath the body results in increased comfort and may prevent hypothermia which is important for injured and sick patients in ambulance care.

## Introduction

Prehospital patients are vulnerable to cold exposure especially in situations of grave danger such as serious injury situations or severe illnesses [1]. Hypothermia is an independent predictor of increased morbidity and mortality regardless of injury severity [2]. Several studies state the physiological effects of hypothermia [3]–[5]. Factors that may impair the body's natural thermoregulatory mechanisms are fatigue, central or peripheral nervous system injuries, medication, and the influence of alcohol or drugs [5],[6]. Additionally, age, female gender, chronic illness, trauma, starvation, and endocrine diseases [5],[7] are regarded as aggravating circumstances. Traditionally, hypothermia has been defined as body core temperature <35°C. Because of poor prognosis in the combination of trauma and hypothermia the level has been set to <36°C [8].

A review study described hypothermia as initiated by cold exposure and peripheral vasoconstriction leading to peripheral blood being shunted to the central body regions in order to support the vital organs and to retain heat [9]. Initially, the cold is experienced and observed in the hands and feet, but the back also cools quickly [1]. In general, after being cold, it is difficult to get warm and it takes longer to recover than it does to become cold [6]. Although there are major shifts in temperature in the environment, the human body strives for a constant temperature between 36–38°C, which is the temperature at which physiological functions operate optimally [9],[10].

Patients can be expected to have a negative experience even before the body reaches a temperature low enough to qualify as hypothermia [11]. Aléx et al. [12], showed in their study that patients injured outdoors in cold environments experienced anxiety of freezing to death. Despite injury coldness gradually became the primary problem. They expressed that it felt good to receive active heat supply and warmth spreading throughout their whole body.

A review study by Kober, Scheck, Fulesdi, Lieba, Vlach, Friedman and Sessler [4], showed increased anxiety and perceptions of pain in participants when exposed to cold. After the patients had been wrapped with a warm blanket, they stressed that they experienced decreased anxiety and had a more positive experience because it increased the thermal comfort. Similar results were seen in elderly patients who experienced an external heat supply to be positive. The heat supply increased satisfaction and decreased experience of fear and restlessness [13],[14].

In nursing, preventing thermal discomfort can be seen as an important way to satisfy fundamental needs [15]. Thermal comfort seems to improve patients’ psychological and physiological status and is also important for prehospital patients. From a nursing perspective warmth seems to contribute to experiences of comfort and safety. In prehospital settings there are studies concerning the supply of heat sources such as chemical heating pads, hot water bottles, charcoal heater [16], hot air blankets, and reflective blankets on top of the body [4],[5]. Prehospital rewarming with heated intravenous fluids is not efficient and intravenous fluids cool rapidly and aggravate hypothermia [5],[17]. In prehospital care, in Sweden, warm intravenous fluids are available, but if the ambient temperature is lower than 37°C the intravenous fluids will adapt to the ambient temperature and instead contribute to a decreasing body temperature [17]. Polyester blankets used in ambulance care have a bad insulation value in windy conditions compared to indoor use [18].

Despite these findings, limitations exist concerning equipment for reducing risks causing decreased thermal comfort for patients in modern ambulances. There is a lack of research about the effect of heat supply from underneath the body. We assume that it would be beneficial to prevent conductive heat loss by using a heated mattress underneath the patient because of the back’s large surface.

Exposure to cold temperatures is often a neglected problem in prehospital care [1]. Cooling of the back is one of the leading influences of the overall sensation of discomfort from cold [19]. Exposure to a cold environment is by far, the largest contributor to hypothermia [9]. Thus, in prehospital care, it is important for nursing staff to identify and prevent heat loss to avoid patients’ thermal discomfort and hypothermia [1],[12],[20].

In the wintertime, when the stretcher or equipment is taken out of the ambulance, the patient compartment and associated equipment is cooled down quickly, and the patients are also exposed to cold by conduction [11]. Conduction is the most effective method of heat and cold transfer [17].

It seems important to investigate the effect on thermal comfort using active heat supply from underneath. Can a heated mattress have a positive impact on body temperatures and increase thermal comfort for persons requiring ambulance transport? The aim of this study, therefore, was to evaluate effects of a heated ambulance mattress-prototype on body temperatures and thermal comfort in an experimental study.

## Method

### Design

Both quantitative and qualitative data were used to obtain a more comprehensive picture than what either method would reveal used alone [21].

### Setting

Data were collected during four days in November, 2011 inside and outside of a cold chamber. The cold chamber had a constant temperature (+2°C). Outside the cold chamber, the temperature was approximately +21°C. Data were collected by three ambulance nurses. Each nurse cared for one participant at a time. In the cold chamber, each nurse had a separate area from the other nurses when collecting data from the participants. The stretchers were in different rooms and the participants had no possibility to communicate with each other.

### Participants

Twenty-three of approximately 50 students from the Umea University Nursing Department agreed to participate in the experiment, 20 women and three men. The average participant age was 24, varying between 19 and 46. Exclusion criteria were being pregnant, smoking or snuff user, having cold-induced asthma, and being medicated with circulation affecting drugs. None of the 23 students were excluded.

### Experimental procedure

**Figure 1:**
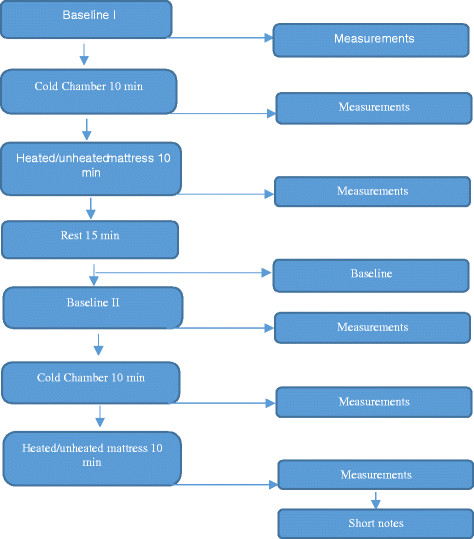
**Observation schedule.** Two trials, in and outside the cold chamber.

All participants participated in two trials each. In one trial, they were lying on a stretcher supplied with a heated mattress and in the other trial without a heated mattress. Three stretchers were used, of which one had a heated mattress. The participants performed the experiment three at a time with an ambulance nurse each of their side during the experiment. The first trial began with measurements (Figure 1) at room temperature, i.e., baseline. Participants were then exposed to cold temperature in the cold chamber for 10 minutes. The participants were told to wear only one layer of clothing, i.e., trousers and a thin shirt, and to stand up without moving their hanging arms. The individual measurements were repeated after 10 minutes in the cold chamber. They then left the cold chamber and lay down in a room with 21°C on an ambulance stretcher with a sheet and wrapped with a blanket. They were randomly selected to lie down on an ambulance stretcher supplied with a heated mattress (n = 8) or without a heated mattress (n = 15). The heated mattress is 150 cm long and is connected to 12 volts with a temperature of + 30°C. After ten minutes on the stretcher, a third measurement was performed. Thereafter, the participants had 15 minutes to recover their body temperature with warm clothes until they felt that they returned to their original temperature at baseline (Figure 1). The second trial was identical except that the participants now used the opposite stretcher, either supplied with a heated mattress or no heated mattress.

At each measurement time, temperatures were measured, back temperature between the shoulders and finger temperature, on outer fingertips, left hand were measured with an IR thermometer with dual laser points indicating the measurement area, CIR 8819. Measurements were taken from approximately 7 cm from the measurement surface/area (3.5 cm Ø). Core body temperature was measured via internal ear, infrared light (IR), with a Braun Thermo Scan, Exac Temp IRT 4520, Germany.

To measure thermal discomfort, Cold Discomfort Scale (CDS) was used; it has been used in previous studies [7],[22],[23] and validated [24]. The scale ranges from 0–10; 0 is equal to no discomfort from the cold, and 10 is the worst possible discomfort from the cold. Further, four statements from the STAI [25] were used. The statements “I am tense”, “I feel comfortable”, “I am relaxed”, and “I feel content” were measured. The answers to the four statements from the STAI instrument were estimated from 1–4 where 1 = Not at all, 2 = Somewhat, 3 = Moderately So, and 4 = Very Much So [25]. At the end of the day, the participants (n = 21) wrote down short notes about their experiences of laying on the different mattresses. No structured questions were asked in the notes section.

### Quantitative data analysis

Sample size calculation showed that a minimum of 17 participants was required in each trial. The endpoint finger temperature was assumed to differ 0.5°C between the trials with a power of 80% and a significant level of 5%. To examine differences between trials, the data were separated according to the heated mattress trial (n = 23) and the unheated mattress trial (n = 23). Paired-Samples *t*-Test and Wilcoxon signed rank test were used. The difference between having spent 10 minutes in the cold chamber and then lying on the mattresses 10 minutes was calculated on an individual level for each parameter/statement. Difference in mean, standard deviation, significance level, and effect size were calculated. The statistical analyses were performed with SPSS software (version 18.0, SPSS Inc., Chicago IL, USA).

### Qualitative data analysis

The participants were asked to make their own short notes at the end of the experiment. The short notes, including a few sentences per participant, were analysed according to manifest qualitative content analysis [26], where the short notes were categorized in four categories, corresponding to the aim of the study.

In the present study, the qualitative and quantitative data were analysed separately. Using qualitative and quantitative datasets may provide greater insight than would have been possible only considering each dataset separately [21].

### Ethical considerations

The study was approved by the Regional Ethical Review Board in Umeå, (reference number 2011-343-31 M). Participation was voluntary, and participants were informed both orally and written about the experimental approach, and that they could withdraw from the study at any time. No one outside the research team had access to the material. All material from the observations was treated confidentially.

## Results

### Quantitative results

A statistically significant increase and a large effect size were seen in back temperature between the heated mattress trial compared to the unheated mattress trial. The difference concerning finger temperature, ear temperature, and CDS showed no statistical significance (See Table 1.)

**Table 1:** Difference in mean* of back temperature, cold discomfort scale (CDS), ear and finger temperatures between the heated mattress trial (n = 23) and the unheated mattress trial (n = 23)

	**Unheated mattress trial**	**Heated mattress trial**			
	**Mean (SD)**	**Mean (SD)**	**Difference in mean (%)**	**p-value**	**ES**
**Back temperature (°C)**	12.0 (2.98)	14.4 (3.70)	2.4 (20.0)	0.009 ˜ ˜	0.27 ˜
**Ear temperature (°C)**	−0.05 (0.42)	−0.01 (0.34)	0.04 (80)	0.69 ˜ ˜	0.00 ˜
**Finger temperature (°C)**	8.9 (5.15)	8.2 (5.31)	0.7 (8)	0.867††	0.35 †
**Cold Discomfort Scale (CDS)**	−3.5 (1.73)	−4.2 (1.78)	0.7 (20.0)	0.063††	0.38†

*Difference between having spent 10 minutes in the cold room and then lying on the stretcher 10 minutes. A negative value denotes a decrease.

˜ ˜From Paired-Sample *t*-Test.

˜Effect Size (Eta Squared) 0.01 = small or no effect, 0.06 = moderate effect, 0.14 large effect.

††From Wilcoxon signed-rank test.

†Effect Size (Cohen) 0.1 = small effect, 0.3 = medium effect, 0.5 large effect.

There was a statistically significant decrease in the participants rating of the statement “I am tense” (p = 0.011, ES = 0.37) from the heated mattress trial compared to the unheated mattress trial; a medium effect size. There was a statistically significant increase and medium effect size of the participants’ statements “I feel comfortable” (p = 0.012, ES = 0.37), “I am relaxed” (p = 0.029, ES = 0.32), and “I feel content” (p = 0.008, ES = 0.39) from the heated mattress trial compared to the unheated trial.

### Qualitative results

The heated mattress was positively experienced. The analysis resulted in four categories. The analysis from text to category, exemplified with quotations, is described in Table 2.

**Table 2:** Examples of findings from the qualitative data

**Area**	**Categories**	**Citations**
The heated mattress	Being warm and comfortable	“The stretcher felt immediately warm and the heat spread out through the body. After a short while the body was warm again”, “The heated mattress felt much warmer even in the neck.”
	Provides security	“I felt secure when embedded on the stretcher.”
The unheated mattress	Being unrelaxed	“It was more difficult to relax on the unheated mattress.”
	Not being warm	“It felt good to have a blanket but the heat took time to return.”

1. *Being warm and comfortable.* The experiences of the heated mattress were expressed as warm and pleasant, especially on thighs and back, and laying on the heated mattress was a good experience and the whole body was warmed up.

2. *Provides security.* It was a good experience to be on the heated mattress. It was expressed that the heated mattress transferred heat and safety.

3. *Being unrelaxed.* It was expressed that it was difficult to relax on the unheated mattress because of the cold, and that it was cold to lie on.

4. *Not being warm.* It was expressed that it took a long time to get warm on the unheated mattress, and some participants did not feel any warmth for a long time afterwards.

## Discussion

The aim of this study was to evaluate effects of a heated ambulance mattress-prototype on body temperatures and thermal comfort in an experimental study. It has been a contentious issue whether hypothermic patients should be warmed or not in prehospital care. The justification against heating has been a risk of complications, but there is also a lack of equipment and monitoring methods to control the effect. Increasingly, studies [3],[12],[16],[27]–[29] show positive results using different active warming methods already in the ambulance. It has become clear that this is a safe and profitable complement to other medical interventions. However none of the previous studies have investigated active heat delivered from underneath the body.

Several studies from hospital settings have investigated different methods to prevent and treat cold exposure, e.g., warm blankets [13],[14]. Warming polyester blankets has been shown to be ineffective and the initial benefit dissipated in about 10 minutes [30].

In the present study, the participants show that the heated ambulance mattress-prototype had an effect on thermal comfort. The result show that the participants had a higher back temperature, higher rating on the CDS, and felt less tense, more relaxed, more comfortable and more content in the heated mattress trial compared to the unheated mattress trial.

The back temperatures were higher in the heated mattress trial compared to the unheated trial. There is a lack of research of active heat delivered from underneath the body in prehospital care; however, there are intraoperative studies stating that using an underbody warming system is effective in preventing hypothermia [31],[32]. We argue that a constant active heat source is preferable to no active heat source in prehospital care. A chemical heating pad supplied on the chest to prehospital patients showed a more positive experience, compared to only supplying blankets [12],[22]. As in our study, similar results using warm blankets in operation units showed increased comfort and reduced anxiety [4]. Sessler and Schroeder [30] described how 90% of persons in cold environments who were given an electric blanket on top of the body experienced it to be comfortable. Electric blankets were also shown to more effectively reduce acute low back pain for patients being transported to hospital when compared to woolen blankets [33]. Results from our previous study show that the unheated ambulance mattress appears to be an important factor in cooling the patients. The patients seem to warm up the stretcher, and not the other way around [11]. We argue that the supply of active heat is important because the synthetic blankets used in prehospital care that patients are wrapped in, are almost worthless in windy conditions as 8 m/sec [18] and in environments with cold temperatures [11]. We also argue that when using heat from underneath, the staff has better access to examine the patient compared to when using resistive blankets.

Participants in the present study showed no significant decrease on the estimate of the CDS scale in the heated mattress trial compared to the unheated mattress trial. However, the qualitative result showed that the heated mattress felt much warmer compared to the unheated mattress.

The statements showed a decrease of the statement "I'm tense" and an increase on the statements "I feel comfortable”, “I am relaxed”, and “I feel content”. The statements can be seen as increased thermal comfort associated with the heated mattress. Candas and Dufour [34] clearly state that there is a relationship between skin temperature and discomfort. Cooling of the back is one of the leading influences of the overall sensation of discomfort from cold [19]. Similar to our study, other studies states that active heating during ambulance transport increases thermal comfort, core temperature, reduces pain and anxiety, and improves overall patient satisfaction in prehospital care [4],[14],[22]. Following the results from our study and other research results [3],[16],[27]–[29], we argue that it is important to provide active heat in prehospital care, such as in the ambulance and that thermal comfort might contribute to the possibility for the patient to rest in a stressed situation. Our study on a heated mattress has shown positive results on thermal comfort.

The short notes from the participants showed that they experienced the heated mattress to be warm, safe, and easier to relax on. They found it pleasant to be warm on thighs and back, and they expressed laying on a heated mattress as a good experience and that the whole body was warmed. This result provides a more comprehensive picture to the quantitative findings.

There were no significant decreases on ear temperature. The temperature effect (30°C) of the heated mattress is probably too low to increase body core temperature in 10 minutes. The mattress is, however, intended to be used on all patients, aiming to prevent thermal discomfort and to avoid contact with cold materials for sick and injured patients. When caring for hypothermic patients we recommend using the heated mattress in combination with other active heat sources.

### Methodological considerations

In the present study, we found it was useful to use both quantitative and qualitative methods to provide a more comprehensive picture, which also is highlighted by others [35].

The cold chamber had a constant temperature (+2°C), which meant that all participants were exposed to the same temperature. Therefore, variations due to situational factors such as wind, snow, and rain were eliminated. The time in the cold chamber and on the stretchers was constant for all participants. The same type of equipment and devices were used as in an ordinary ambulance care, and all participants wore the same type of clothing during the experiment. We consider the study and the measurement accuracy and reliability to be high in terms of choice of method and relevance and that validity of the study is good.

We are aware that most students in the nursing department are women. Women have, in general a lower hand temperature and are more sensitive to cold exposure by air temperature [36]. However, in the present study we have focused on the differences between the two mattress types and not on gender differences. Data were collected by three ambulance nurses, and even with a careful approach before the study, there is still a risk that the measures were collected differently. However, we believe it could be more of a strength because this data collection is more controlled and more reliable with its clear instructions and few researchers when compared with measurements performed in patient care, e.g., by various ambulance nurses. The significance between trials may have been larger if using a larger sample size or if the time in the cold chamber had been extended. Still, we believe that the results and significant effect size have clinical importance.

The participants considered themselves to be healthy without the influence of trauma, disease, alcohol, or medication. With the exclusion criteria, we considered that some factors that affect reactions to cold were eliminated. This means that our conclusions can only be generalized to that specific group. Despite this, we believe the results are transferable to prehospital care because the experiment was set in controlled conditions without influences of fear and pain that can influence the vasoconstriction and the feeling of chill/cold; and the results show an effect of active heat. Thermal comfort for injured and unhealthy patients can be different from healthy people because of the physical disability that will affect thermophysiology, thermal sensation, blood flow metabolism regulatory response as vasomotor control of body skin temperature, or the ability to sweat [37]. Injured or sick patients may have a worse experience of thermal discomfort in a decreased temperature compared to young and healthy students.

### Clinical implication and future research

Future research is needed on patients in ambulance care. Other studies together with present results are evidences enough for expediting the process of clinical implementation with active heat supply in ambulance care.

## Conclusion

As shown in this study, using a heated mattress after being exposed to cold increases thermal comfort. There are several positive aspects of using active heat from underneath in the form of mattresses for use in today's ambulances. Heat supply from underneath the body results in increased comfort and may prevent hypothermia which is important for injured and sick patients in ambulance care.

## Competing interests

The authors declare that they have no competing interest.

## Authors’ contributions

JA: Planning the study, data collection, analysis and writing of the manuscript. SK: Analysis and supervising of the manuscript. B-IS: Supervising and planning the study, analysis and writing of the manuscript. All authors have participated in the manuscript according to the criteria for authors. All authors read and approved the final manuscript.

## Authors’ information

JA, RN, PEN, MSc, PhD student

B-I S, RNT, PhD, professor

SK, RN, PhD, professor
